# A Systematic Self-Certification Model for Mobile Medical Apps

**DOI:** 10.2196/jmir.2446

**Published:** 2013-04-24

**Authors:** Thomas Lorchan Lewis

**Affiliations:** ^1^Warwick Medical SchoolUniversity of WarwickCoventryUnited Kingdom

**Keywords:** smartphone, technology, education, medicine, app, health care, Android, iPhone, BlackBerry, mobile phone, standards

Errol Ozdalga and colleagues recently highlighted the impressive range of roles and uses of smartphones in the medical setting [1]. An important point highlighted and worth developing from this paper is the difficulties associated with accurately comparing and assessing different medical apps for smartphones. This is mainly due to the fact that medical apps are often designed with one particular focus and inherently different interfaces which often make a direct comparison between apps unfeasible. Furthermore, even apps that purport to complete the same task often include extra functionality or features that make direct evaluation impossible. One solution offered by Ozdalga et al is to survey doctors on the perceived impact of specific apps available. However I believe that this is unfeasible given the rate at which the medical app ecosystem is evolving in terms of number, range, and type of app. With thousands of medical apps available, it is highly improbable that a clinician has a working knowledge of the complete range available. As such, any surveys will be subjective depending on the target audience and consequently offer limited utility for physicians and medical students alike. Moreover, surveys regarding specific apps are usually out of date by the time they are published. What is more important, is establishing a systematic method by which medical apps can be compared and their utility for health care professionals validated.

 

One proposed method to solve this is to develop a set of standard criteria that can be used to systematically assess the utility of a medical app for a health care professional. I believe that the most efficient and effective method should be based on a self-certification system with key criteria that have been adapted from the Health on the Net foundation (HON, [2]). Table 1 shows potential self-certification criteria which medical apps could be reasonably expected to achieve in order to establish the validity of the information contained within the app. The Health on the Net Foundation Code of Conduct (HONcode) for medical and health websites addresses one of Internet's main health care issues: the reliability and credibility of information. It is therefore highly applicable to medical apps that are subject to the same issues.

 

Using this system, it would then possible to set up a self-certification process where registered developers could highlight the fact that their app conforms to these basic criteria. At the moment, no such organization exists although there is clearly scope for such an entity. With the impending launch of the United Kingdom National Health Service App Store, it appears that there has never been a better time to develop a self-certification model for medical apps.

**Table 1:** A list of potential criteria based on the HONcode to be used as the basis of a self-certification model for medical apps.

Certification criteria	Detailed description
Information must be authoritative	All medical information presented in a medical app must be attributed to an author and his/her training in the field must be mentioned.
Purpose of the website	A statement clearly declaring that the information on the app is not meant to replace the advice of a health professional has to be provided. A brief description of the app’s mission, purpose, and intended audience is necessary. Another brief description of the organization behind the app, its mission, and its purpose is also necessary.
Confidentiality	This principle is applicable to all apps, even if it does not host patient records or store any medical or personal data. The app must describe a privacy policy regarding how confidential, private or semi-private information such as email addresses and the content of emails received from or sent to users is treated. Users must be informed whether their data will be recorded in your own database, who can access this database (others, only you, nobody), if this information is used for your own statistics (anonymous or not), or if these statistics are used by third party or other companies. Even if one or more of these points are not relevant to your app, you must state how you handle the following information sent to you by your visitors: (email addresses or/and contact information, names, personal, or medical data).
Information must be documented: referenced and dated	All medical content (page or article) has to have a specific date of creation and a last modification date. All sources of the medical content must be clearly indicated the recognized, scientific, or official sources of health information quoted in the app. Ideally, a precise link to the source is provided whenever it is possible.
Justification of claims	All information about the benefits or performance of any treatment (medical and/or surgical), commercial product, or service is considered as claims. All claims have to be backed up with scientific evidence (medical journals, reports, or others).
Contact details	The app must be completely operational and the information must be accessible and clearly presented. There must be a way to contact the developer, such as a working email address or contact form, for users who would like to have more details or support. This contact must be easy to access from anywhere within the app.
Financial disclosure	Each app must include a statement declaring its sources of funding. This is required for all apps, including those with no external sources of funds, and apps funded by government agencies, pharmaceutical companies, or other commercial entities. All funding must be declared: government agency, private companies, donations, etc. Developers also have to declare all conflicts of interest.
Advertising policy	Conflicts of interest and external influences which could affect the objectivity of the editorial content must be clearly stated in a disclaimer. All apps displaying paying banners have to have an advertising policy. This policy must explain how the app distinguishes between editorial and advertising content and which advertisements are accepted. Any conflict of interest has to be explained.
